# Evolving pneumococcal serotypes and sequence types in relation to high antibiotic stress and conditional pneumococcal immunization

**DOI:** 10.1038/srep15843

**Published:** 2015-11-02

**Authors:** Lin-Hui Su, An-Jing Kuo, Ju-Hsin Chia, Hsin-Chieh Li, Tsu-Lan Wu, Ye Feng, Cheng-Hsun Chiu

**Affiliations:** 1 Department of Laboratory Medicine, Chang Gung Memorial Hospital, Taoyuan, 333, Taiwan; 2 Chang Gung University College of Medicine, Taoyuan, 333, Taiwan; 3 Molecular Infectious Disease Research Center, Chang Gung Memorial Hospital, Taoyuan, 333, Taiwan; 4 Institute for Translational Medicine, Zhejiang University School of Medicine, Hangzhou, China; 5 Department of Pediatrics, Chang Gung Memorial Hospital, Taoyuan, 333, Taiwan

## Abstract

In Taiwan, beginning in 2013, the 13-valent pneumococcal conjugate vaccine (PCV13) was provided free of charge to children 2–5 years of age. In 2014, this was extended to children 1–5 years old. During 2012–2014, 953 cases of culture-confirmed pneumococcal disease (CCPD), including 104 invasive pneumococcal disease (IPD), were prospectively identified and analyzed at a 3,700-bed hospital in Taiwan. From 2012 to 2014, the incidence per 10,000 admissions decreased from 26.7 to 20.4 for CCPD (*P* < 0.001) and from 3.2 to 1.9 for IPD (*P* < 0.05). Significant reduction of PCV13 serotypes was firstly noted in children in 2013 and extended to both paediatric and adult populations in 2014. Simultaneously, the incidence per 10,000 admissions of non-PCV13 serotypes increased from 6.1 in 2012 to 9.3 in 2014 (*P* < 0.005). The most prevalent non-PCV13 serotypes were 15A, 15B, and 23A, each containing a predominant clone, ST63^15A^, ST83^15B^, and ST338^23A^. From 2012 to 2014, isolates with penicillin minimum inhibitory concentrations >2 mg/L decreased from 27.8% to 8.1% (*P* < 0.001) among all isolates. PCV13 immunization in young children demonstrated an early protective effect in all ages. However, in the elderly, the effect was compromised by an emergence of non-PCV13 serotypes.

*Streptococcus pneumoniae* is among the leading causes of bacterial pneumonia, meningitis, and sepsis and is associated with high morbidity and mortality. According to a report from the World Health Organization (WHO), *S. pneumoniae* is responsible for 1.6 million deaths annually, particularly among young children and the elderly^1^. Increased antimicrobial use has led to the increase of antimicrobial resistance in *S. pneumoniae*, adding the difficulty in the management of pneumococcal infections^2^. Vaccines were therefore developed for the prevention of pneumococcal infections^3^.

The first pneumococcal conjugate vaccine (PCV) was the 7-valent PCV (PCV7), licensed in the United States (US) in 2000^4^. This vaccine contains pneumococcal serotypes 4, 6B, 9V, 14, 18, 19F, and 23F. Universal immunization of children with the PCV7 not only reduced the incidence of pneumococcal infections in children, but also led to an indirect effect in the elderly, resulting in an overall decline of invasive pneumococcal disease (IPD)^5^. However, infections caused by non-vaccine serotypes, 19A in particular, increased significantly^5^,^6^. A 10-valent PCV (PCV10) containing the serotypes in PCV7 plus serotypes 1, 5, and 7F was developed subsequently. Another 13-valent PCV (PCV13) containing the serotypes in PCV7 plus serotypes 1, 3, 5, 6A, 7F, and 19A was further developed. PCV13 was licensed by the Food and Drug Administration (FDA) in the United States for use in children in 2010, and later for adults aged over 50 years in late 2011^7^. PCV13 now has been recommended by the Advisory Committee on Immunization Practices (ACIP) for routine use among adults aged over 65 years^8^.

Taiwan is associated with a high prevalence of penicillin-resistant pneumococci^9^, partly due to the widespread use of antimicrobial agents^10^. Serotypes 19F, 23F, 6B, 14, and 3 had been the most common among all or invasive pneumococcal isolates before PCV7 was introduced for private use in 2005^11^,^12^. Soon after, an increasing number of serotype 19A infections were reported^13^,^14^,^15^. PCV10 was introduced in 2010, but was found adding little benefit as the 3 extra serotypes have been rare in Taiwan^13^,^14^,^15^. PCV13 was introduced into Taiwan in 2011 also in the private sector. Since March 2013, a national catch-up immunization with PCV13 has been launched for all children 2–5 years old who did not receive any PCV13^16^. In 2014, the lower age of eligibility for the campaign was reduced to 1year of age. By the end of 2014, the age-appropriate immunization rate has reached ~80% according to the report from the Centers for Disease Control in Taiwan. To provide timely and continuous surveillance information, the present study was initiated in 2012. This hospital-based longitudinal study reflects the changing pneumococcal epidemiology in the context of high antibiotic selection pressure and conditional use of PCV13 in Taiwan.

## Materials and Methods

### Setting and bacteria

The present study was conducted between January 2012 and December 2014 at Chang Gung Memorial Hospital. With a vast capacity of 3,700 beds, this university-affiliated hospital is the largest hospital in Taiwan and serves patients from the whole Taiwan district, including the main and scattered islands. No apparent or significant change regarding patient admission and/or management has been made in recent years. During the study period, no apparent outbreak of upper respiratory tract infections, including seasonal flu, has been noted in Taiwan.

Clinical isolates of *S. pneumoniae*, one isolate per patient, were prospectively collected. All isolates were cultured and identified with standard methods^17^. An IPD case was defined as isolation of *S. pneumoniae* from a normally sterile body site. The annual admission numbers of patients in this hospital were 135,013 in 2012, 133,377 in 2013, and 138,756 in 2014. The annual incidence of culture-confirmed pneumococcal disease (CCPD) was calculated and expressed as the number of cases/10,000 admissions. IPD was included in CCPD which referred to the simultaneous presence of any type of diseases and the isolation of pneumococci from the associated clinical specimens. For sputum culture, the quality of specimens were confirmed before the culture was proceeded, and only the predominant micro-organisms were reported.

### Antimicrobial susceptibility

Minimum inhibitory concentrations (MICs) of penicillin and ceftriaxone were examined by E-test strips (bioMérieux, Marcy l’Etoile, France). The Clinical and Laboratory Standards Institute (CLSI) has recommended different MIC interpretive criteria for meningeal and non-meningeal infections^15^. In the present study, for the consistency of data analysis, antimicrobial susceptibility results were categorised as susceptible (S; penicillin, ≤0.06 mg/L; ceftriaxone, ≤0.5 mg/L), reduced susceptible (RS; penicillin, 0.12-2 mg/L; ceftriaxone, 1 mg/L), and non-susceptible (NS; penicillin, >2 mg/L; ceftriaxone, >1 mg/L). According to the CLSI criteria, susceptibility in the RS category indicated that the *β*-lactam remained effective for non-meningeal infections but should be interpreted as resistant for meningeal infections^18^.

### Serotyping and multi-locus sequence typing (MLST)

Serotypes of the pneumococcal isolates were determined by using commercialised antisera (Statens Serum Institut, Copenhagen, Denmark) and polymerase chain reaction (PCR) methods^19^,^20^. MLST was further determined by PCR-sequencing of a set of pneumococcal house-keeping genes (*aroE*, *gdh*, *gki*, *recP*, *spi*, *xpt* and *ddl*)^21^. The sequence data were compared to the MLST database maintained in the public domain (http://pubmlst.org/spneumoniae/). New alleles and allelic profiles were submitted to the database curator for the assignment of ST numbers. eBURST analysis was used to group sequence types (STs) into clonal complexes (STs shared six of the seven MLST loci).

### Statistical analysis

The Chi-square test or Fisher’s exact test, when appropriate, was used. A difference was considered statistically significant with a two-tailed *P* < 0.05.

## Results

### Incidence rates and serotype distribution

**Figure 1:**
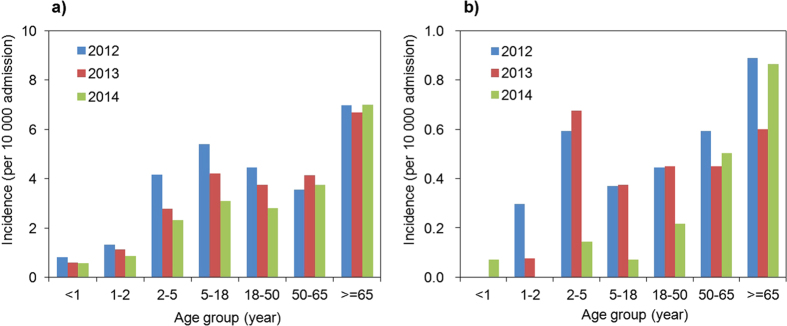
Changes in estimated age-specific incidence of (**a**) pneumococcal disease and (**b**) invasive pneumococcal disease during 2012–2014.

**Figure 2:**
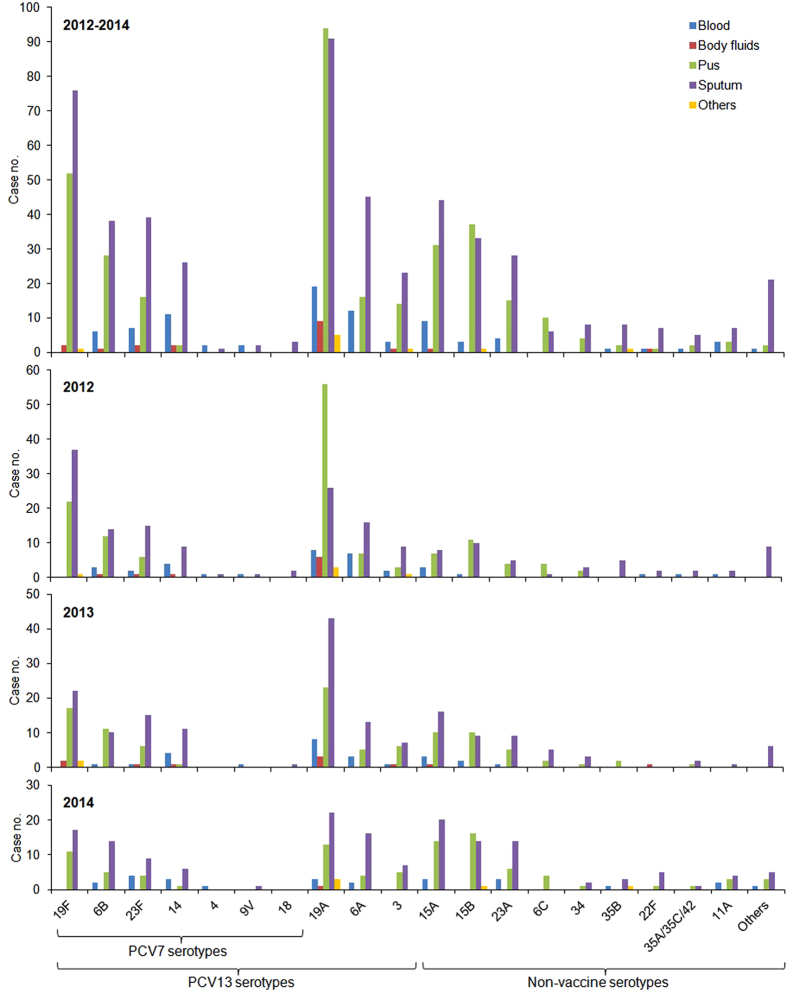
Site of infection-related serotype distribution among the 953 pneumococcal isolates studied during 2012–2014. [Other serotypes are 13, 20 (*n* = 4 each), 10A (*n* = 3), 8, 16F, 17F, 37 (*n* = 2 each), 7B/7C, 15F, 21, 22A, and 23B (*n* = 1 each)].

A total of 953 CCPD cases (360 in 2012, 310 in 2013, and 283 in 2014) were analysed. The annual incidence per 10,000 admissions decreased from 26.7 in 2012 to 20.4 in 2014 (*P* < 0.001). Among them, 104 (10.9%) were IPD cases, including 5 (0.5%) cases of meningitis. The incidence per 10,000 admissions of IPD cases decreased from 3.2 in 2012 to 1.9 in 2014 (P < 0.05). As shown in Fig. 1, the decline was found mainly in the age groups between 2–50 years old in both IPD and non-IPD cases. The isolates were identified from sputum and other respiratory samples (53.6%), pus (34.5%), blood (8.9%), body fluids (2.0%), and others (1.0%) (Fig. 2). The majority (87.7%) of the respiratory isolates were from patients ≥8 years old with clinical presentations of pulmonary infections. In contrast, pus isolates were mostly (69.6%) identified from ear discharge specimens of infants or pre-school children suffering acute otitis media with otorrhoea.

A total of 29 serotypes were identified with 9 isolates remained undetermined (serotypes 35A/35C/42, *n* = 8; serotypes 7B/7C, *n* = 1; Fig. 2). The most prevalent serotypes were 19A and 19F. However, the proportion of serotype 19A decreased from 27.5% in 2012 to 14.8% in 2014 (*P* < 0.0005). The reduction was most significant among the pus isolates (41.8% in 2012 vs. 14.1% in 2014, *P* < 0.0001; Fig. 2) and in the paediatric age groups <18 years old (44.9% in 2012 vs. 20.0% in 2014; *P* < 0.0001; Supplementary Fig. S1). Serotype 19F isolates also decreased from 16.7% in 2012 to 9.9% in 2014 (*P* < 0.05). The decrease was similar among different age groups during the study years (Fig. 2 and Supplementary Fig. S1).

Among the IPD cases (Fig. 2 and Supplementary Fig. S2), serotype 19A (26.9%) was still the most prevalent serotype in each study year, and its proportion was highest in the age group of 2-5 years old (73.7%). Serotype 14 (12.5%) was the second most prevalent serotype and was most commonly found in patients over 65 years old (31.3%). Serotype 6A (11.5%) was the third most prevalent serotype and was most frequently found in the age group of 50–65 years old (28.6%). These major serotypes all decreased in 2014, and in each age group, the case number was only 2 or less (Supplementary Fig. S2).

Overall, the incidence per 10,000 admissions of PCV13 serotypes decreased from 20.6 in 2012 to 16.5 in 2013 (*P* < 0.05) and to 11.1 in 2014 (*P* < 0.0001). The significant decrease was found in both paediatric (from 9.4 to 3.3; *P* < 0.0001) and adult (from 11.2 to 7.8; *P* < 0.005) groups from 2012 to 2014. However, in 2013, the significant decrease was found only in the paediatric age groups (from 9.4 to 6.5 per 10,000 admissions; *P* < 0.001).

In contrast, the incidence per 10,000 admissions of non-PCV13 serotypes increased from 6.1 in 2012 to 9.3 in 2014 (*P* < 0.005). The increase was observed in both paediatric (from 2.3 to 3.5 per 10,000 admissions; *P* = 0.0587) and adult (from 3.8 to 5.8 per 10,000 admissions; *P* < 0.05) groups from 2012 to 2014 (Supplementary Fig. S1). The most predominant non-PCV13 serotypes were 15A, 15B, and 23A (Fig. 2). The incidence per 10,000 admissions of these serotypes all increased from 1.3, 1.6, and 0.7 in 2012 to 2.7 (*P* < 0.05), 2.2 (*P* = 0.2556), and 1.7 (*P* < 0.05) in 2014, respectively, becoming similar to the incidence rates of serotypes 19A (3.0 per 10,000 admissions) and 19F (2.0 per 10,000 admissions) (Fig. 2).

### *β*-Lactam susceptibility

For ceftriaxone, the rates of isolates that were not susceptible (NS) or of reduced susceptibility (RS) were 38.2% and 32.4%, respectively. The NS rates were significantly higher in the paediatric age groups (46.6%) than in the adult age groups (32.9%; *P* < 0.0001), whereas the RS rates were similar (paediatric, 34.4%; adult, 31.2%; *P* = 0.2960). From 2012 to 2014, the NS rates in the paediatric age groups decreased from 51.9% to 37.9% (*P* < 0.05), whereas the RS rates increased from 32.9% to 40.0% (*P* = 0.2541). The changes were not found among the adult age groups.

For penicillin, the rates of isolates that were not susceptible (NS) or of reduced susceptibility (RS) were 15.0% and 74.2%, respectively, during the study period. However, from 2012 to 2014, the NS rates actually decreased significantly from 27.8% to 8.1% (*P* < 0.0001), whereas the RS rates increased significantly from 61.9% to 79.5% (*P* < 0.0001). The significant change from NS toward RS during the study period was observed in both paediatric and adult groups. Still, throughout the study years, the overall NS rates remained significantly higher in the paediatric age groups (20.1%) than in the adult age groups (11.8%; *P* < 0.001), whereas the RS rates were similar (paediatric, 75.6%; adult, 73.3%; *P* = 0.4249). A high proportion (73.9%) of the penicillin-NS isolates collected in 2014 belonged to PCV13 serotypes.

**Figure 3:**
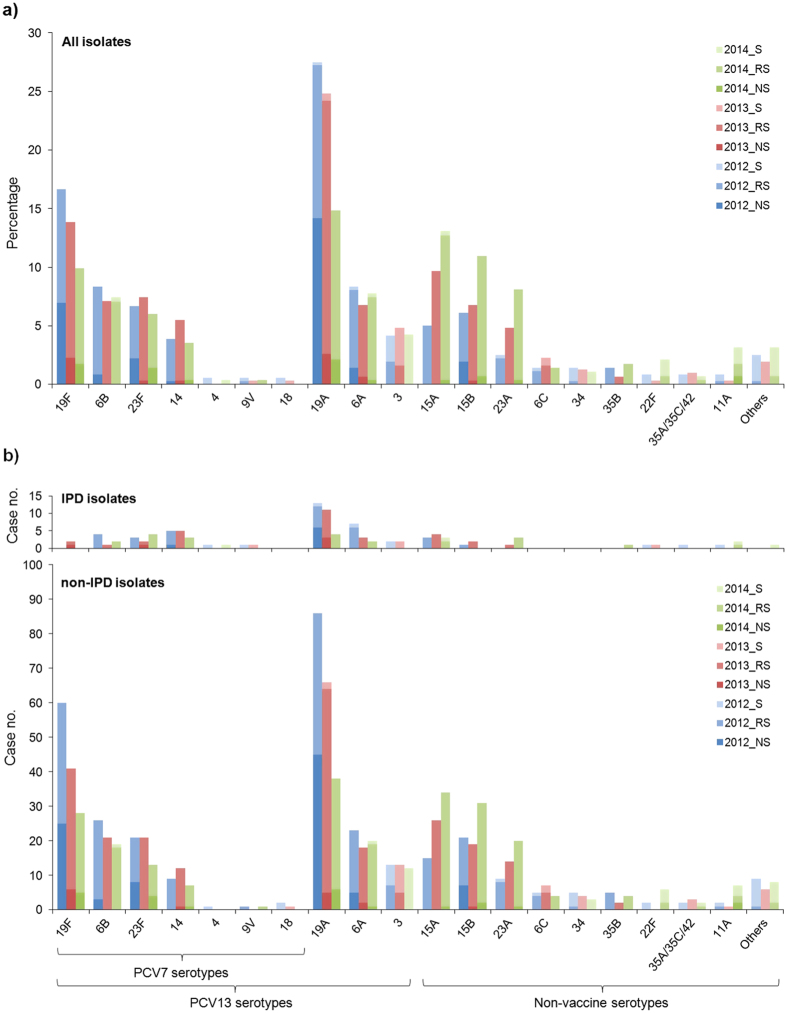
Penicillin susceptibility among (**a**) all isolates and (**b**) invasive pneumococcal disease (IPD)-related isolates (upper) and non-IPD isolates (lower). Panel (**a**) is expressed in accumulated percentages using the respective annual total isolate number as the denominator. (S, susceptible, ≤0.06 mg/L; RS, reduced susceptible, 0.06-2 mg/L; NS, non-susceptible, >2 mg/L) (Other serotypes are as those listed in Fig. 2).

Serotypes 19A and 19F demonstrated the highest penicillin NS rates, both of which decreased significantly from 51.5% and 41.7% in 2012 to 10.4% and 16.3% in 2013, and to 14.3% and 17.9% in 2014, respectively (*P* < 0.05). Simultaneously, the penicillin RS rates increased from 47.5% and 58.3% in 2012 to 87.0% and 83.7% in 2013, and to 85.7% and 82.1% in 2014, respectively (*P* < 0.05; Fig. 3a). Such a significant shift from penicillin NS toward RS in 2013-14 was also observed in serotypes 23F and 15B isolates from the adult age groups (*P* < 0.05). The 3 major non-PCV13 serotypes, 15A (97.6%), 15B (86.5%), and 23A (95.7%), all showed a high RS rate during the study period (Fig. 3a). In contrast, the other minor non-PCV13 serotypes demonstrated a significantly lower penicillin RS rate (37.9%; *P* < 0.0001). Penicillin NS was found in only two serotype 11A isolates.

For IPD cases (Fig. 3b), the penicillin NS was found in the prevalent serotypes, such as 19A, 14, 19F, and 23F, and none in the non-PCV13 serotypes. The 5 isolates causing meningitis belonged to 4 different serotypes (3, 19F, 22F, and 23F). *β*-Lactam resistance (MIC, 2–4 mg/L for both penicillin and ceftriaxone) was found in serotypes 23F (*n* = 2) and 19F (*n* = 1).

### Sequence types and serotypes

A total of 158 sequence types were identified during the study period, and 74 were new types first reported in this study (Table 1 and Supplementary Fig. S3). Four major STs were found in almost half of the isolates studied: ST320 (22.5%), ST81 (8.9%), ST63 (8.6%), and ST83 (6.1%). ST320 and the closely related ST236^22^, together with ST271, ST1464, and other 17 minor STs, formed the largest clonal complex CC236/320 (*n* = 332, 34.8%), which included the majority of isolates from serotypes 19A (*n* = 211, 96.8%) and 19F (*n* = 119, 90.8%). Another large clonal complex CC81/83 (*n* = 159, 16.7%) included most of the isolates from serotypes 6A (*n* = 55, 75.3%), 15B (*n* = 59, 79.7%), and 23F (*n* = 34, 53.1%). The third clonal complex was CC63 (*n* = 86, 9.0%), mostly belonging to serotype 15A (*n* = 82, 96.5%). The fourth clonal complex was CC338 (*n* = 41, 4.3%), mostly belonging to serotype 23A (*n* = 38, 80.9%). Some minor clonal complexes, such as CC242 (*n* = 28, 2.9%), CC876 (*n* = 22, 2.3%), CC2652 (*n* = 14, 1.5%), and CC7130 (*n* = 18, 1.9%), were noted (Table 1 and Supplementary Fig. S3). They included a substantial part of the isolates in serotypes 23F (*n* = 25, 39.1%), 14 (CC876, *n* = 22, 53.7%; CC2652, *n* = 13, 31.7%), and 6B (*n* = 18, 24.7%), respectively (Table 1). Prevalent STs in some serotypes were also found: ST180 in serotype 3 (95.2%), ST717 in serotype 35A/35C/42 (*n* = 7, 87.5%), ST433 in serotype 22F (*n* = 7, 70.0%), ST99 in serotype 11A (*n* = 6, 66.7%), and ST76 in serotype 6B (*n* = 41, 56.2%) (Table 1). From 2012 to 2014, significant changes were found in the proportion of CC236/320 (decreased from 42.5% to 23.0%; *P* < 0.0001), CC63 (mainly ST63, increased from 3.9% to 10.0%; *P* < 0.001), and CC338 (mainly ST338, increased from 1.6% to 5.1%; *P* < 0.005).

Among the 43 global clones currently designated by the Pneumococcal Molecular Epidemiology Network (PMEN) (http://spneumoniae.mlst.net/pmen/pmen.asp)^23^, 6 were identified in the present study: Sweden^15A^-25 (*n* = 79), Netherlands^3^-31 (*n* = 40), Taiwan^19F^-14 (*n* = 32), Spain^23F^-1 (*n* = 28), Taiwan^23F^-15 (*n* = 21), and Colombia^23F^-26 (*n* = 1) (Table 1). However, isolates from other serotypes could also share the same STs with these PMEN global clones, including ST81 (Spain^23F^-1) found in most (*n* = 49, 67.1%) serotype 6A isolates and sporadic isolates of serotypes 19F, 6B, and 6C (Table 1). Most (*n* = 35, 74.5%) of the serotype 23A isolates belonged to ST338, the same as Colombia^23F^-26. Two serotype 6C isolates also belonged to ST242, the same as Taiwan^23F^-15 (Table 1).

Compared to isolates from adult patients (122 STs), those from children (69 STs; Supplementary Fig. S3) were relatively less diverse. A total of 33 STs were found among the IPD isolates, and the clonal complex CC236/320 remained the largest group (*n* = 29, 30.5%; Supplementary Fig. S3), although ST236 was not found among the IPD isolates.

## Discussion

In Taiwan, the incidence of IPD is highest in children aged 2–5 years^13^,^15^,^16^. We found in the present study a significant reduction of IPD, particularly in young children. Although this is only a hospital-based observation, our results were in-lined with those observed in the real-time monitoring system hosted by the Centers for Disease Control in Taiwan (http://nidss.cdc.gov.tw/en). The phenomenon is likely attributable to the national catch-up campaign for PCV13 among children under 5 years of age. Our study also revealed that non-IPD diseases, particularly those caused by PCV13 serotypes, have reduced significantly both in children and, although later, in adults. We also have noted that the types of pneumococcal diseases may vary in different age groups. Due to the capacity of one single report, further cross analysis regarding the serotype changes among various age groups with various diseases was not performed in the present study. However, the detail analysis remains important from the viewpoints of clinical management of pneumococcal diseases and warrants further studies. Nevertheless, the present report has clearly demonstrated that, despite the suboptimal use of PCV13 in Taiwan, its effectiveness in preventing pneumococcal diseases was evident. A broader adaptation of the PCV13 among children should have the potential to further significantly decrease IPD as 61.5% of the IPD in 2014 was caused by serotypes in PCV13. Incorporation of PCV13 into the national immunization program has shown a profound effect in many countries, with a significant IPD reduction of 40%–70% in total population or the targeted age groups^24^,^25^,^26^,^27^,^28^. The relatively inferior effect observed in the present study due to the emergence of some non-PCV13 clones probably is linked to the conditional immunization policy applied in Taiwan.

As mentioned above, a rapid and significant increase of non-PCV13 serotypes, especially 15A, 15B, and 23A, was noted in the present study. Although these replacement serotypes were predominantly identified from non-IPD cases, significant increase of these serotypes in IPD cases has been reported from England (15A and 23A)^24^, Spain (15A and 23A)^25^, Denmark (15B)^26^, Israeli (15B)^27^, and the US (15A, 15B and 23A)^29^, after the implementation of PCV13. By MLST analysis, we further revealed the clonality of these predominant non-PCV13 serotypes, including ST63^15A^, ST83^15B^, and ST338^23A^, which have not yet been reported. Other than serotype 19A, several pre-existing penicillin NS clones, including ST63^15A^ and ST338^23A^, also increased in the post-PCV7 era^30^. Previous studies showed that ST338 was common in serotype 23A before and after PCV7 introduction in the US^30^,^31^. This clone, ST338^23A^, was also suggested to have emerged in the pre-PCV7 era through capsular switch or structural change from the pre-existing ST338 Colombia^23F^-26 strain^30^,^31^. In Taiwan, serotype 23F had been one of the most prevalent serotypes although information on the ST distribution was very limitied^11^,^12^,^13^,^15^. However, in the present study, we could still find an ST338^23F^ isolate in 2014. All these situations suggested that, similar to what was found in other countries, the capsular switch from ST338^23F^ to ST338^23A^ may have occurred in Taiwan. Actually, serotypes 15A, 15B, and 23A had been identified at low frequency in Taiwan before PCV13 was introduced^11^,^12^,^13^,^14^,^15^. Two ST338^23A^ isolates had been documented in the pre-PCV13 era in Taiwan^12^. In the present study, with the increasing use of PCV13, we observed an increase of ST338^23A^ from 6 cases in 2012, 12 in 2013, to 17 in 2014. Furthermore, earlier reports from Taiwan have demonstrated ST81/ST83 in serotype 23F isolates^11^,^12^. In the present study, we also found 5 isolates of ST83^23F^. Under the selection pressure by PCV13, capsular switch from ST83^23F^ to ST83^15B^ may have occurred. Consequently, while the other predominant serotypes may have been eliminated by PCV13, clonal expansion of these pre-existing minor non-vaccine serotypes, with the advantage of high penicillin RS/NS, could occur and spread widely. Furthermore, spontaneous switch between serotypes 15B and 15C is known to occur at a high frequency in some isolates, although some other isolates may fail to show this reversible change^32^. Subsequent investigations revealed that the genetic difference between the two serotypes lies in the number of a short tandem TA repeat in the capsular polysaccharide synthesis (*cps*) locus, and the reversible switch may be strain dependent^33^. Apparently, the serotype 15B isolates in the present study belonged to those that could not exhibit the spontaneous switch, and therefore we did not find any serotype 15C isolate in this study.

Some emerging serotypes, such as serotypes 6C^24^,^25^, 10A^26^, 11A^24^, 8, 20^24^,^26^, 22F^24^,^26^,^28^, and 23B^26^,^29^, found in the European countries, and serotypes 21, 23B^29^, 22F^28^, 35B^29^,^34^, found in the US, were only sporadically identified in the present study. The lower penicillin RS/NS rates found among these minor non-PCV13 serotypes may partly explain their less predominance in the present study. With the increasing use of PCV13, other changes in the serotype replacement phenomenon may occur. However, it seems that emerging non-PCV serotypes after PCV13 use were more diverse than those observed in the post-PCV7 era. Although remained to be clarified, the mechanism behind this observation may have involved some spatial and temporal factors. If the regional diversity persisted, the design of future conjugate vaccines covering more serotypes may be difficult, and other vaccine targets should be considered^35^,^36^.

Several PMEN-defined global antibiotic-resistant clones were identified in the present study. Except the non-PCV13 serotype 15A, the other clones have been prevalent in Taiwan^11^,^12^. This finding suggests that various international antibiotic-resistant clones have been co-circulating in this area. Among the major CCs, Taiwan^19F^-14 was included in the largest CC236/320, which consisted of 34.8% of the total pneumococcal isolates studied. Other than the original Taiwan^19F^-14 clone ST236, 22 other closely related STs were also found. The observation is similar to reports from other countries where a known PMEN global clone may invade, propagate, and evolve to become a dominant clone in the local area^37^,^38^,^39^. ST320^19A^, the most common clone in the present study, was shown to be genetically derived from Taiwan^19F^-14 (ST236) and became a better coloniser in the nasopharynx^22^. By using whole genome sequencing analysis, a recent study also provided evidence for the success of this vaccine-escaping lineage as a result of soft selective sweeps during the evolution of pneumococcal multidrug resistance^40^. The rapid diversification through homologous recombination observed in the global collection of *S. pneumoniae* may have occurred under the suboptimal pneumococcal immunization and antibiotic selective pressure in Taiwan.

In countering pneumococcal disease, the use of PCVs has been the closest weapon to a triumph. The efficacy and sustainability of the protection effect by PCVs has been evidenced by several long-term studies around the world^24^,^25^,^26^,^27^,^28^. However, gaps in PCV introductions were still noted in Asia and in countries with large birth cohorts; only 31% of the world’s birth cohort currently has access to PCV. WHO recommendations for use and financial support through the Gavi Alliance for PCV introduction in lower-income countries likely contributed to the increased use of PCV. Middle-income countries, including Taiwan, that are not eligible for Gavi Alliance support need to weigh vaccine procurement and operational costs against costs of other health priorities. Results in the present study revealed the impact of conditional PCV13 vaccination in a middle-income country with high antibiotic selective pressure. Although the overall pneumococcal disease burden has been reduced, some replacement serotypes with low *β*-lactam susceptibilities have been emerging, especially in the elderly population. To maximize the effect of vaccination, judicious antimicrobial usage to reduce resistance selection pressure is essential. The inclusion of PCV13 in the compulsory immunization program, not only in young children, but also in those aged 65 years and older, as has been recommended by the US ACIP in 2014^8^, may help the overall control of pneumococcal disease.

## Additional Information

**How to cite this article**: Su, L.-H. *et al.* Evolving pneumococcal serotypes and sequence types in relation to high antibiotic stress and conditional pneumococcal immunization. *Sci. Rep.* **5**, 15843; doi: 10.1038/srep15843 (2015).

## Supplementary Material

Supplementary Information

## Figures and Tables

**Table 1:** Sequence types of various serotypes among the 953 clinical pneumococcal isolates.

Sequence type orclonal complex (CC)	Total	Year			PCV7/PCV10 serotypes							PCV13 serotypes			Non-vaccine serotypes			
		2012	2013	2014	19F	6B	23F	14	4	9V	18	19A	6A	3	15A	15B	23A	Others^a^
Total	953	360	310	283	131	73	64	41	3	4	3	218	73	42	85	74	47	95
CC236/320																		
320	214	99	75	40	11		1					202						
271	38	13	15	10	37							1						
236	32	18	8	6	32^b^													
1464	18	7	9	2	17											1		
283	6	3	1	2	6													
1465	5	2	1	2	5													
7122	5	4		1								5						
Others^c^	14	7	5	2	11							3						
CC81/83																		
81	85	33	30	22	4	3	28^d^						49					1
83	58	21	17	20			5					1				52		
4003	2	1		1									2					
282	2	1	1										1					1
8528	2	1		1												2		
Others^e^	10	2	2	6			1						3		1	5		
CC63																		
63	82	17	28	37	1	1							1		79^f^			
Others^g^	4	1	2	1				1							3			
CC338																		
338	38	7	12	19	1		1^h^								1		35	
8080	2		1	1													2	
2777	1	1															1	
CC242																		
242	23	7	12	4			21^i^											2
Others^j^	5	3	1	1			4							1				
CC876																		
876	18	8	6	4				18										
5749	3	1	1	1				3										
9126	1		1					1										
CC2652																		
2652	10	2	5	3	1			9										
13	2		2					2										
15	2	2						2										
CC7130																		
7130	10	6	3	1		10												
90	3			3		3												
8526	3	2		1		3												
95	2	1		1									2					
Others^k^	2	1	1			2												
180	40	15	13	12										40^l^				
76	43	15	16	12		41							2					
166	11	4		7						3							3	5
Others^m^	157	55	42	60	5	10	3	5	3	1	3	6	13	1	1	14	6	86

Abbreviations: PCV7, 7-valent pneumococcal conjugate vaccine; PCV10, 10-valent pneumococcal conjugate vaccine; PCV13, 13-valent pneumococcal conjugate vaccine.

^a^Other minor serotypes were 6C (*n* = 12), 34 (*n* = 12), 35B (*n* = 12), 22F (*n* = 10), 11A (*n* = 9), 35A/35C/42 (*n* = 8), 20 (*n* = 4), 10A (*n* = 3), 13 (*n* = 3), 8, 16F, 17F, 37 (*n* = 2 each), 7B/7C, 15F, 21, 22A, and 23B (*n* = 1 each).

^b^PMEN global clone Taiwan^19F^-14.

^c^ST2697, ST3164, ST7107, ST7123, ST8525, ST8537, ST9121, ST9122, ST9124, ST9129, ST9135, ST9136, ST9630, and ST9998 (*n* = 1 each).

^d^PMEN global clone Spain^23F^-1.

^e^ST8524, ST9117, ST9118, ST9131, ST9229, ST9990, ST9991, ST10072, ST10077, and ST10079 (*n* = 1 each).

^f^PMEN global clone Sweden^15A^-25.

^g^ST782, ST7340, ST8019, and ST9639 (*n* = 1 each).

^h^PMEN global clone Colombia^23F^-26.

^i^PMEN global clone Taiwan^23F^-15.

^j^ST1435, ST1441, ST9116, ST9994, and ST9625 (*n* = 1 each).

^k^ST9123 and ST9133 (*n* = 1 each).

^l^PMEN global clone Netherlands^3^-31.

^m^Other STs included 93 minor STs, each consisted of 1-8 isolates.
