# Supplementary material for: Evolving pneumococcal serotypes and sequence types in relation to high antibiotic stress and conditional pneumococcal immunization
Source: Sci Rep. 2015 Nov 2;5:15843. doi: 10.1038/srep15843 (PMC4629140; doi:10.1038/srep15843)
Supplement: Supplementary Information [file srep15843-s1.pdf]

Evolving pneumococcal serotypes and sequence types in relation to high antibiotic stress and conditional pneumococcal immunization

Lin-Hui Su, An-Jing Kuo, Ju-Hsin Chia, Hsin-Chieh Li, Tsu-Lan Wu, Ye Feng, and Cheng-Hsun Chiu

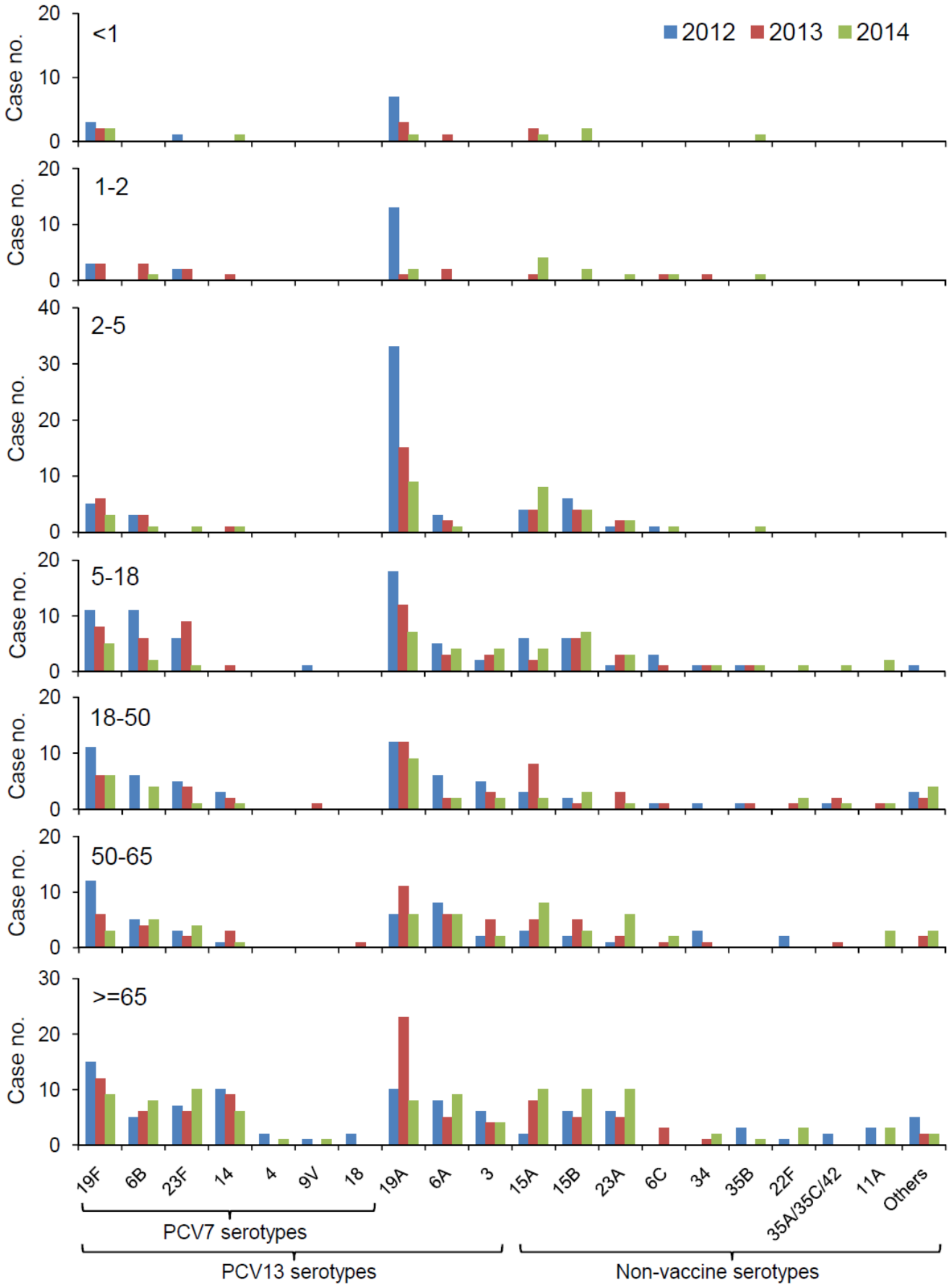

Supplementary Figure S1. Age-specific serotype distribution among the 953 pneumococcal isolates studied during 2012-2014. [Other serotypes are 7B/7C, 8, 10A, 13, 15F, 16F, 17F, 20, 21, 22A, 23B, and 37].

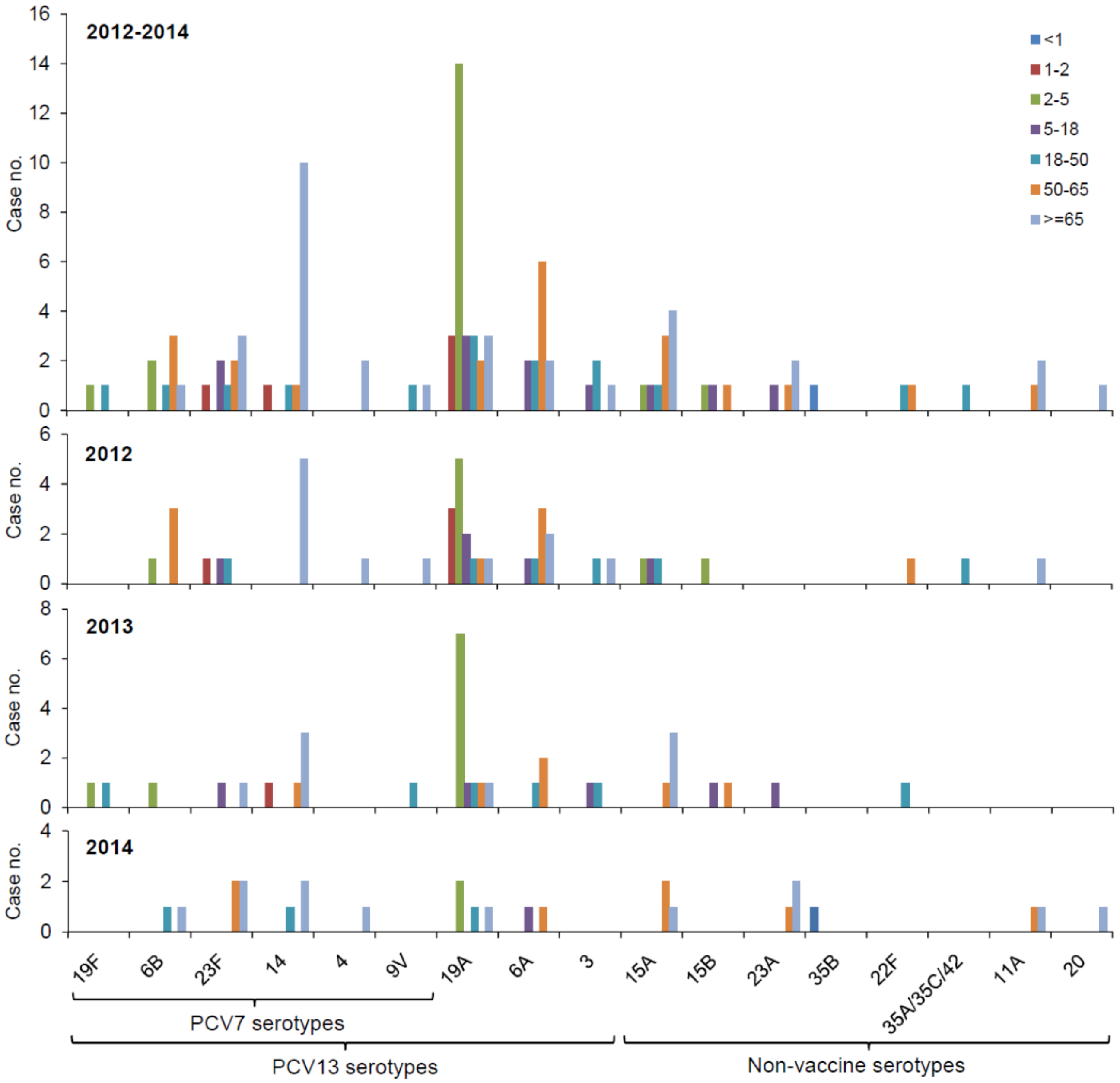

Supplementary Figure S2. Age-specific serotype distribution among the 104 invasive pneumococcal disease cases during 2012-2014.

a)

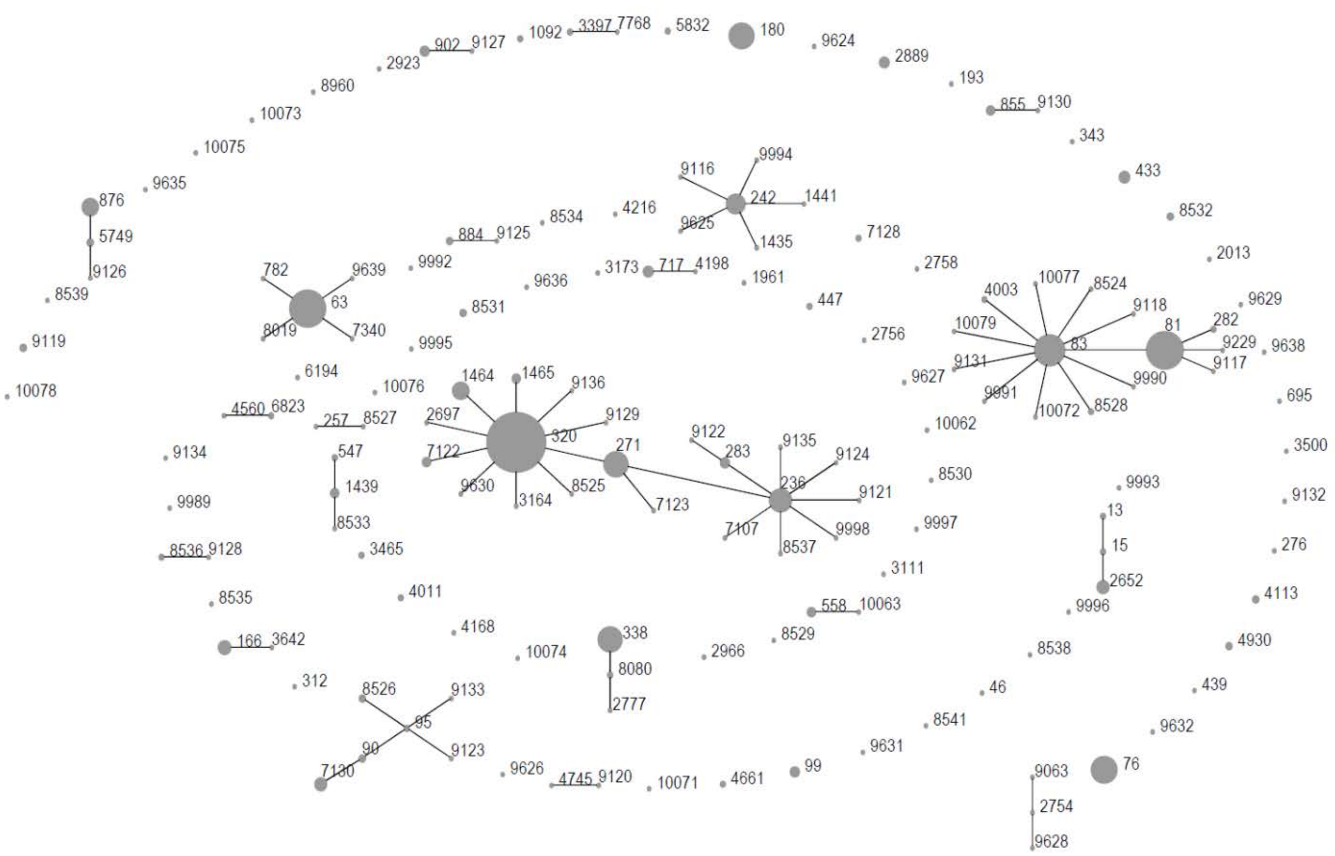

b)

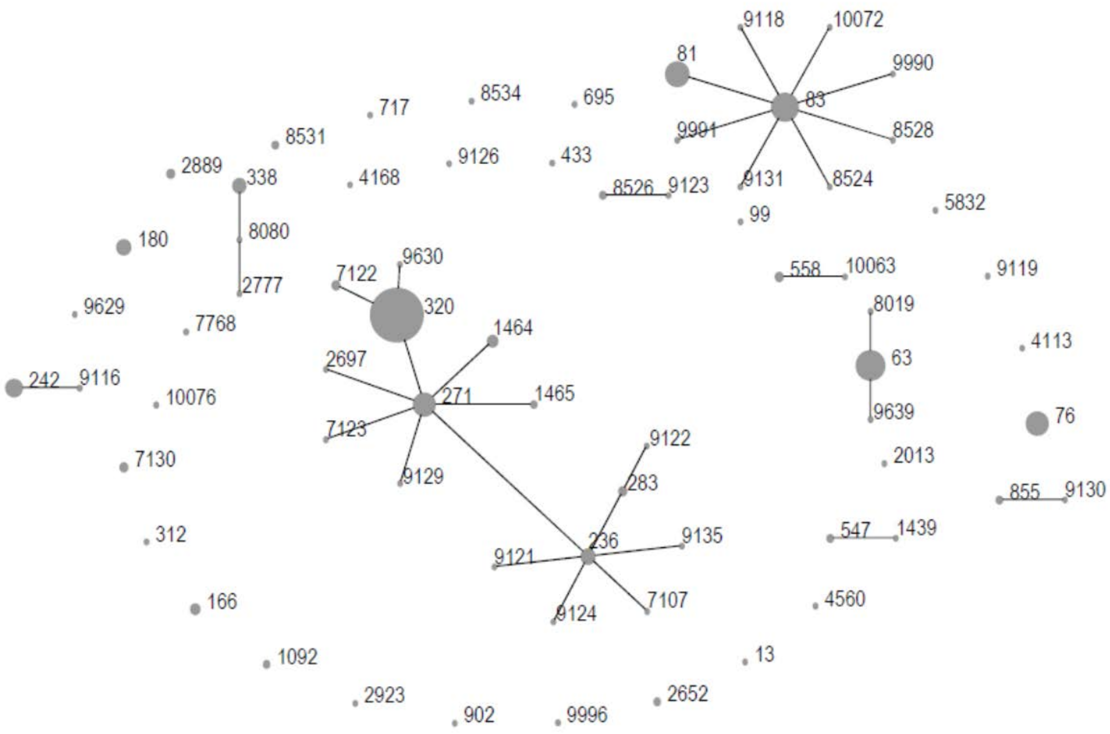

c)

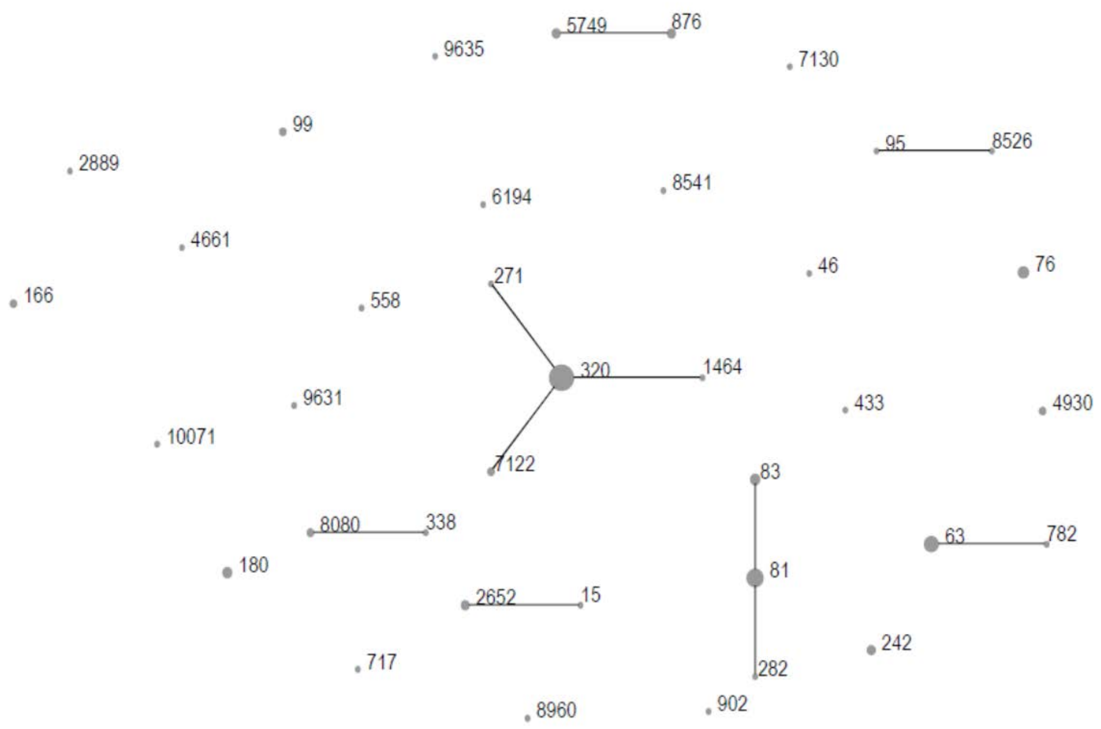

Supplementary Figure S3. Population snapshot of a) the 953 pneumococcal isolates, b) the 369 isolates from patients < 18 years of age, and c) the 104 invasive pneumococcal disease-causing isolates revealed by eBURST analysis. One dot represents a single sequence type (ST) and the number is indicated. The size of the dot corresponds to the number of isolates in each ST. The linkage between two dots indicates the two STs are single locus variants for each other.
